# Clinical and Epidemiological Determinants of Lower Respiratory Tract Infections in Hospitalized Pediatric Patients

**DOI:** 10.1155/2020/8844420

**Published:** 2020-11-17

**Authors:** Mohammadreza Mirkarimi, Mohsen Alisamir, Salar Saraf, Solmaz Heidari, Shahriar Barouti, Shooka Mohammadi

**Affiliations:** Department of Pediatrics, Faculty of Medicine, Ahvaz Jundishapur University of Medical Sciences, Ahvaz, Iran

## Abstract

**Background:**

Lower respiratory tract infection (LRTI) is the main cause of pediatric mortality and morbidity in low- and middle-income countries.

**Purpose:**

This study was carried out to determine the clinical and epidemiological characteristics of children with LRTI.

**Method:**

A retrospective study was conducted on all pediatric patients who were hospitalized due to LRTI in Abuzar Hospital (Ahvaz, Iran) during one year. Incomplete medical records and children who were treated on an outpatient basis, as well as infants younger than 1 month of age, were excluded. The patients were evaluated in terms of epidemiological, clinical, and paraclinical characteristics.

**Results:**

A total of 303 hospitalized children and infants were identified. Their mean age was 29.09 ± 38.96 months (range 1 month-15 years), and 59.4% of them were males. The highest frequency of patients was at the age below one year (50.8%, *n* = 154). Pneumonia and bronchitis were the most common LRTIs. Respiratory (54.6%) and neurological (21.6%) diseases were the most prevalent underlying medical conditions. Admission was more common in winter (*n* = 120, 39.6%) and spring (*n* = 79, 26.1%). The mean length of stay (LOS) in the hospital was 8.2 ± 5.5 days, and the overall mortality rate was 11.6%. In addition, 65 patients were severely underweight and 271 patients were malnourished. Moreover, there was a significant association between mortality and disease diagnosis (*p* < 0.05). Furthermore, there was a significant association between having an underlying disease and consanguineous parents (*p* < 0.01), as well as the frequency of hospitalization (*p* < 0.001).

**Conclusion:**

Additional studies are required to determine factors contributing to disease severity among children with LRTI to develop appropriate preventive and therapeutic strategies.

## 1. Introduction

Acute respiratory tract infection (ARI) is the major cause of death and morbidity in low- and middle-income countries (LMICs) [1, 2] in all age groups and genders [3]. ARIs are generally caused by viruses or bacteria, which are categorized into lower respiratory tract infections (LRTIs) and upper respiratory tract infections (URTIs) [4]. LRTIs refer to any infection involving the respiratory tract below the level of the larynx, which include bronchiolitis, bronchitis, tracheitis, and bronchopneumonia [2, 5]. They are determined by the occurrence of symptoms and signs of acute respiratory infection (cough, oropharyngeal hyperemia, and nasal discharge) and lower respiratory signs (retraction, extended expiratory time, tachypnea, and wheezing or crackles on auscultation) [6].

The majority (97%) of acute lower respiratory infection (ALRI) cases are found in LMICs [7], where 6.9 million children died in 2011 and nearly one in five of these deaths were due to ALRI [8]. In 2016, LRTIs caused 652,572 deaths in children under the age of five, worldwide [9]. Particularly in developing countries, they are critical public health problems in the early years of life with almost one-fifth of all deaths among children younger than five years old [10–12].

Bronchiolitis among acute LRTIs is the major common cause for hospitalization of infants [13]; however, pneumonia is the most common cause of death in adults (mainly above 65 years old) and children, comprising 3.46 million (6.1%) deaths globally and over 1.05 million (11.3%) in lower-income countries [14, 15]. In 2008, pneumonia was identified in almost 156 million children (5 and 151 million in developed and developing countries, respectively) [16]. Of the 156 million new cases of pneumonia among children per year, more than 20 million patients with severe disease are admitted annually to hospitals [16, 17]. Community-acquired pneumonia (CAP) is a typical and possibly dangerous infection in childhood, with a declining occurrence as children grow up [18]. In 2010, the incidence of childhood CAP in LMICs was around 0.22 annual incidence per child, with 11.5% of cases of severe disease [19].

Risk factors related to the environment have a major impact on the occurrence of pediatric respiratory tract infections [2]. The most important risk factors are nonexclusive breastfeeding, malnutrition, low birth weight, absence of measles vaccination in children under age one [2], zinc and vitamin A deficiency, concomitant diseases (e.g., asthma, heart disease, and diarrhoea), low level of the mother's education, poor socioeconomic status, indoor crowding, air pollution, parental smoking, humidity, and cold weather [2, 16, 20]. Enhancements in the nutritional status of children, supportive care, improved access to antibiotics, and increased vaccination uptake have decreased the frequency of deaths due to LRIs in many countries [21].

ARIs in children constitute a large burden of diseases which require health care; therefore, it is necessary to determine the exact epidemiological and clinical data of ARIs for health policymakers to produce and implement rational strategies for the management of these diseases according to local evidence-based guidelines and improve patient quality of care [22]. Several studies have been conducted in Iran on selected populations of children with ARI in different regions of the country with diverse results [22]. At present, inadequate data are available related to pediatric LRTIs in Iran as a middle-income country. To advance the public health insights into the interrelated burdens of childhood LRTI in Iran, we have critically checked the available clinical and epidemiological data of pediatric patients with LRTI who admitted to a hospital in southwest Iran.

## 2. Materials and Methods

This retrospective epidemiological study was carried out among all pediatric patients who were admitted due to LRTI in Abuzar Hospital (Ahvaz, Iran) during one year (July 2017-July 2018). The Medical Ethics Committee of Ahvaz Jundishapur University of Medical Sciences (AJUMS) approved the current study (IR.AJUMS.REC.1397.300). The patients were in the age range from one month to 15 years. The medical records of 368 children and infants were evaluated. The documents of 65 patients were excluded from the study. Finally, a total of 303 children (180 boys and 123 girls) were included in this study. The diagnostic criteria at the time of admission were clinical symptoms (fever, cough, and changes in breath sounds) and radiography of the lungs (infiltration, consolidation). The study involved children diagnosed with LRTI symptoms (initial symptoms or final diagnosis), including pneumonia, bronchiolitis, and pertussis-like syndrome (PLI). The exclusion criteria were children who were treated on an outpatient basis (outpatient diagnosis and treatment), those with incomplete medical records, and infants younger than one month of age. After preparation of the checklist, all pediatric patients' medical records over the 1-year duration were reviewed to extract the required data. Patients' data, including gender, age, body temperature, clinical symptoms, admission time, preclinical findings, hospitalization period (days), vital signs, height, weight, nutritional status (using standard *Z*-scores), mortality rate, underlying disease, and final diagnosis, were summarized for this study. In addition, vaccination coverage was assessed with the immunization card. Complete immunization was taken as age-appropriate immunization according to the expanded program of immunization (EPI) schedule in Iran. Partial immunization was defined as incompletion of the EPI schedule. A nonimmunized child was defined as not receiving any vaccination. The consistency and completeness of the records were cross-checked and extracted in Excel software.

### 2.1. Statistical Analysis

All data were analyzed using the Statistical Package for the Social Sciences (SPSS) version 25 (SPSS Inc., Illinois, USA). The Kolmogorov–Smirnov test was applied to identify the normality of data. Descriptive data were reported as percentage, frequency, median (interquartile range), and mean (standard deviation). The continuous variables were compared using the independent *t*-test. The chi-squared (*χ*^2^) test was applied to find any associations between the categorical variables. The associations between independent and dependent variables were also evaluated using linear and logistic regression tests (odds ratio with 95% confidence interval). The *p* < 0.05 was considered the level of significance.

## 3. Results

In the current study, a total number of 303 medical records of hospitalized children and infants with LRTI between 2017 and 2018 were reviewed. The majority of patients were males (59.4%, *n* = 180), and 40.6% (*n* = 123) were females, while there was no significant association between gender and LRTI. The mean age was 29.09 ± 38.96 months (ranging from one month to 15 years), and there were no statistically significant differences in the mean age between the two genders. The highest rates of hospitalization for severe LRTI and mortality during LRTI hospitalization were in children under age one (50.8%, *n* = 154) and decreased with age (Table 1).

The mean weight and height for boys and girls are displayed in Table 2. Of the 303 patients, 65 (21.5%) and 33 (10.9%) patients were severely (<−3 *Z*-score) and moderately underweight. In addition, 54 (17.8%) and 37 (12.2%) patients were severely (<−3 *Z*-score) and moderately stunted. There were no significant differences in weight and height (cm) between the two genders.

The patients had presented to the hospital averagely 7.50 ± 12.39 days after the onset of symptoms (range 1 day-90 days). Admission was more common in winter (*n* = 120, 39.6%) and spring (*n* = 79, 26.1%) compared with fall (*n* = 60, 19.8%) and summer (*n* = 44, 14.5%). The mean body temperature of children at the time of admission was 37. 55°C (range 35.6-40°C). In addition, 11.2% of patients (*n* = 34) were born preterm. The most common symptoms were cough (*n* = 234, 77.2%), tachypnea (*n* = 171; 56.4%), fever (*n* = 154, 50.8%), and tachycardia (*n* = 115, 37.9%) at the time of admission. Furthermore, all children were fully immunized, and age-appropriate vaccinations were completed according to the expanded program of immunization (EPI) schedule in Iran.

Raised ESR and leukocytosis were found in 261 and 88 patients, respectively. In addition, 66 patients were neutrophil predominant. A C-reactive protein (CRP) test had been performed and was increased in 150 of the cases. Abnormal CRP levels were significantly associated with longer length of stay (LOS) in hospital (*p* < 0.05).

Pneumonia (*n* = 226, 74.6%) and bronchitis (*n* = 52, 17.2%) were the most common LRTIs among patients, and there was no significant association between gender and type of LRTI disease (Table 3). There was a significant trend, based on the logistic regression test, towards a month increase in their age and lower risk of pneumonia and bronchiolitis in the patients (*p* < 0.001).

The mean length of hospital stay was 8.2 ± 5.5 days (range 1-57), and the overall mortality rate was 11.6% (*n* = 35). The mortality rates caused by pneumonia and bronchitis were 10.23% (*n* = 31) and 0.66% (*n* = 2), while there was a significant association between mortality and disease diagnosis (*χ*^2^ = 22.37, <0.05) (Table 3). In addition, there was no significant association between the LOS and mortality rate.

Approximately more than half of the children (61.05%, *n* = 185) hospitalized with LRTI had at least one underlying medical condition and 142 patients had consanguineous parents while 29 patients of this group did not survive (Table 4). In addition, 40 patients required mechanical ventilation while there were 32 deaths among them, and there was a significant association between ventilator support and mortality rate (*χ*^2^211.330, *p* < 0.001).

The most common underlying diseases were respiratory (e.g., asthma, hyperreactive airway disease (HRAD), and cystic fibrosis with pulmonary manifestations) (*n* = 101) and neurological diseases (e.g., spinal muscular atrophy (SMA), polymyalgia rheumatica (PMR), and cerebral palsy (CP)) (*n* = 40). The highest mortality rate was among those with neurological underlying diseases, and there was a significant association between the type of underlying disease and the mortality rate of the patients (*p* < 0.001). In addition, there was a significant association between having an underlying disease and having consanguineous parents (*p* < 0.01). According to linear regression, in patients who did not have a history of the underlying disease, the length of hospital stay was reduced (*β* = −1.5), which indicated a significant association between the underlying disease and length of hospital stay (*p* < 0.05).

Around 32% of patients (*n* = 98) had LRTI hospitalization history while 29% of them (*n* = 87) were hospitalized with LRTI more than one time and nine of them did not survive (Table 4). In addition, there was no significant association between the frequency of hospitalization and mortality rate. Among 118 patients without underlying disease, 115 patients did not have any previous hospitalization and there was a significant association between history of underlying disease and frequency of hospitalization (*p* < 0.001).

## 4. Discussion

In the current study, more than half of the pediatric patients with LRTI were male. The average age of them was around 29 months. The highest LRTI hospitalization rate was among children less than 1 year of age and decreased with age. There was a significant trend towards each month of increase in their age and lower risk of pneumonia and bronchiolitis. However, in a similar study which was conducted in Iran among 182 children, the mean age of patients was 4.7 years and the most common age of the disease was older than 5 years [24]. It has been reported by a similar study in the USA that the rate of hospitalization for severe LRTI declined with increasing age [25]. In another study, among 436 children with ARIs in Nigeria, the most affected age group was 10 to 19 months [20]. This age range is appropriate for the introduction of complementary feeding and reduction of breastfeeding while there are associated risks and weaning of passive maternal antibodies. This may have an impact on the incidence of ARI due to the exposure of these children to the risk factors, which are predominant in this duration. Furthermore, this indicates the role of protective immunoglobulins in breast milk for the prevention of ARIs [20].

In the present study, more boys compared to girls were admitted to the hospital for LRTI, which was similar to other studies that were carried out among children with LRTI hospitalization in the USA [25], Iran [26], Nigeria [20], and China [27]. In this study, there was no significant difference in developing LRTI between boys and girls, which was reported in another study among Iranian children with LRTI [24].

In this study, admission was more common in winter and spring. The seasonal climate is an important factor that can affect pathogen transmission. The difference in seasonal detection maybe was related to a region's climate and demographic factors [28]. In Brazil, 4240 clinical records of children with LRTI were investigated, and the maximum number of cases of pneumonia was found in winter, followed by fall and spring, while lower cases occurred in summer [29]. In another study that was conducted in Turkey to determine the virological characteristics of acute respiratory tract infections (RTIs) among 155 children, most viruses showed strong seasonal patterns. Several viruses were often identified in the rainy and cold seasons [5].

In the current study, pneumonia and bronchitis were the most common LRTIs among patients, which was reported in other similar studies that were performed in South Korea [30] and Nigeria [20]. In our study, the mean LOS in hospital for children with LRTI was 8.2 ± 5.5 days (range 1-57). A systematic analysis revealed that the mean LOS in hospital for children with severe pneumonia was 7.7 (IQR 5.5–9.9) and 5.8 (IQR 5.3–6.4) days in high-income countries (HIC) and LMICs, respectively [31]. LOS in hospital for severe pneumonia was 1.8–4.6 days less in LMICs in comparison to HICs at the median of 6.4 days and a mean of 5.8, which were near to the WHO recommendation of 5-day inpatient treatment [32].

All patients in this study were fully immunized based on completion of age-appropriate vaccinations. In addition, 185 patients had underlying disease, 34 patients were born preterm, and 40 patients required mechanical ventilation. The most common underlying medical conditions were respiratory and neurological diseases. In addition, leukocytosis was present in 88 patients, and increased CRP and ESR were detected in 150 and 261 patients, respectively. In a similar study, among 203 HPIV-positive patients (aged 1 to 71 months) with LRTI in China, a total of 32 (15.8%) had an underlying disease, 21 patients were preterm and 18 patients needed oxygen support, 47 patients had leukocytosis, and 39 patients had increased CRP and ESR [27].

It has been reported that both CRP and ESR had a higher odds ratio in predicting pneumonia than clinical signs alone, and CRP together with clinical signs had adequate sensitivity to estimate the incidence of LRTI [33]. Considerably raised CRP values have typically occurred in patients with pneumonia, and high CRP levels were revealed to be a good predictor of disease in general practice [34]. The increased severity of viral infections has been identified to extend LOS in hospital [35]. Abnormal CRP levels in the current study were associated with longer LOS in hospital. As such, increased CRP levels may be utilized as a factor to predict hospitalization in the intensive care unit or the necessity for mechanical ventilation [36].

The overall mortality rate was 11.6% in this study, which was similar to the mortality rate in another study (12.8%) among 265 patients who were admitted to a PICU in South Africa [37]. In contrast, similar studies among children with LRTI in Mexico and Morocco reported that the mortality rate of children was 1.04% [38] and 4%, respectively [39]. The highest mortality rate in the current study was among those with neurological underlying disease, and there was a significant association between the types of underlying disease and the mortality rate of the patients. In addition, there was a significant association between underlying diseases in children and having consanguineous parents. In patients who did not have any underlying disease, the LOS in the hospital was reduced, which indicated a significant association between the underlying disease and the LOS in hospital.

Around 98 patients had LRTI hospitalization history while 87 patients were hospitalized with LRTI more than one time. There was a significant association between history of underlying disease and frequency of hospitalization. Children with an underlying disease were at an increased risk for severe LRTI. The presence of underlying disease doubled a child's risk for severe LRTI compared with children without any condition, while an increase in the number of underlying diseases increased this risk. Pediatric patients hospitalized with LRTI and equal or more than three underlying diseases had an approximately fivefold greater risk of severe LRTI in comparison with children without any underlying medical conditions [25].

In the present study, 21.5% and 10.9% patients were severely (<−3 *Z*-score) and moderately underweight, respectively. In addition, 17.8% and 12.2% patients were severely (<−3 *Z*-score) and moderately stunted. There were no significant differences in weight and height between the two genders. A similar study in India among 206 children with ALRI (aged from 6 to 60 months) reported that the prevalence of malnutrition among the children was 54.9% [40]. Severe malnutrition was found in 68.7% of children in the 3-5 years age group and 59.4% of them in the 1-3 years age group. In addition, severe malnutrition had a higher frequency in children with severe pneumonia [40]. In a recent study which was conducted in Egypt, among children who were suffering from acute LRTI, there was a positive association between the nutritional status of the child and acute LRTI [41]. There was a significant decrease in mortality and morbidity due to RTI after improvement of the nutritional status of children [42]. The presence of anemia, rickets, and malnutrition were significant risk factors for acute LRTIs and more severe diseases [42].

Recognizing the most cost-effective interventions for LRTI management is critical to achieve the aim of further decreasing childhood mortality. A systematic review verified that early treatment in the community reduced medical costs (per incident) compared with late therapy in the hospital [31]. This finding suggests that the public health community should determine ways for community outreach to access early diagnosis and treatment before the establishment of severe pneumonia [31].

The present study is one of a few single-center studies related to LRTI in Iran. Its findings may improve our insight into the clinical and epidemiological characteristics of children with LRTI in a middle-income and Middle-Eastern country. The main limitation of the study was the retrospective nature of the study. The epidemiology of respiratory viral infections was reported to be varied by geographical region. The prevalence of these viruses in temperate climates is well recognized as a cause of annual winter epidemics of acute LRTIs [43]. The identification of the specific causes of infection prepares a good starting point for the finding of disease attributable to respiratory infection and may provide related data to the development of prevention strategies. These data may reveal important information for program managers and policymakers at regional and national levels to help priority settings, program planning, and resource allocation, as well as to determine the most cost-effective treatment and preventive interventions to decrease the problem of childhood LRTI. Simple approaches should also be considered such as attaining adequate nutritional status in these pediatric patients and educating their parents. However, additional studies are required among children with LRTI to develop appropriate preventive and therapeutic strategies.

## 5. Conclusion

In summary, the highest frequency of patients was at the age younger than one year. Pneumonia and bronchitis were the most common LRTIs, and admission was more common in winter and spring. The mean LOS in the hospital was around 8 days. In addition, the majority of patients were malnourished, and the overall mortality rate was 11.6%. Furthermore, there was a significant association between having an underlying disease and consanguineous parents, as well as the frequency of hospitalization. The findings from this study may reveal important missing data on LRTIs in Iran as a middle-income country. Precise insights into the identification of risk factors for LRTIs, epidemiology, seasonality, and etiology are essential for successful therapy or prevention programs. These risk factors can be modified with simple strategies such as immunization, parental education, adequate nutrition, environmental sanitation, avoidance of pollution, and appropriate counselling of caregivers to the parents. Proper legislation should be used, and health care services should focus on the specific reference to its necessary components such as environmental management, immunization, and nutrition.

## Figures and Tables

**Table 1:** Distribution of children with LRTI according to age and gender (*n* = 303).

Age group	Boys (*n* = 180) (*n* (%))	Girls (*n* = 123) (*n* (%))	Total (*n* = 303) (*n* (%))
<1 year old (1-11 months)	90 (50)	64 (52)	154 (50.8)
1-5 (12-60 months)	58 (32.2)	39 (31.7)	97 (32)
>5 (above 60 months)	32 (17.8)	20 (16.3)	52 (17.2)

**Table 2:** Anthropometric profiles of patients based on gender (*n* = 303).

Anthropometric profile	Boys (*n* = 180) (mean ± SD)	Girls ( = 123) (mean ± SD)
Weight (kg)	10.45 ± 7.13	10.65 ± 7.68
Height (cm)	78.84 ± 25.53	80.45 ± 26.98
WAZ among infant < 2years of age	−1.15 ± 2.08	−0.84 ± 2.09
WAZ among children ≥ 2years of age	−2.30 ± 3.18	−3.03 ± 4.55
HAZ among infant < 2years of age	−1.35 ± 2.84	−0.44 ± 3.89
HAZ among children ≥ 2years of age	−1.02 ± 3.00	−0.95 ± 3.19
Cut off values [23]	(*n* (%))	(*n* (%))
Weigh-for-age *Z*-score		
Severe underweight (<−3.00 WAZ)	41 (22.8)	24 (19.5)
Moderate malnutrition (−3 to −2.01 WAZ)	17 (9.4)	16 (13)
Mild underweight (−2 WAZ to 1.01 WAZ	106 (58.9)	67 (54.5)
Normal (±1.00 WAZ)	16 (8.9)	16 (13)
Height-for-age *Z*-score		
Severe stunting (<−3.00 HAZ)	38 (21.1)	16 (13)
Moderate stunting (−3 to −2.01 HAZ)	25 (13.9)	12 (9.8)
Mild stunting (−2 WAZ to 1.01 WAZ)	90 (50)	66 (53.7)
Normal (±1.00 WAZ)	27 (15)	29 (23.6)

The weight was measured in kilograms. WAZ: weight-for-age *Z*-score; HAZ: height-for-age *Z*-score.

**Table 3:** Distribution of diseases based on the gender and survival of pediatric patients (*n* = 303).

	Pneumonia (*n* = 226) (*n* (%))	Bronchiolitis (*n* = 52) (*n* (%))	Nonspecific LRTI (*n* = 12) (*n* (%))	Others (*n* = 13) (*n* (%))	Total (*n* = 303) (*n* (%))
Gender					
Male	137 (60.6)	28 (53.8)	8 (66.7)	7 (53.8)	180 (59.4)
Female	89 (39.4)	24 (46.2)	4 (33.3)	6 (46.2)	123 (40.6)
Survival status					
Survivors	195 (86.3)	50 (96.2)	12 (100)	11 (84)	268 (88.4)
Nonsurvivors	31 (13.7)	2 (3.8)	0	2 (15.4)	35 (11.6)

**Table 4:** Patients' survival status based on the frequency of hospitalization and underlying disease.

Variables	Survival status		
	Survivors (*n* = 268) (*n* (%))	Nonsurvivors (*n* = 35) (*n* (%))	Total (*n*=303) (*n* (%))
Frequency of hospitalization			
First time	181 (67.5)	24 (68.6)	205 (67.7)
Second time	35 (13.1)	2 (5.7)	37 (12.2)
Third time and more	52 (19.4)	9 (25.7)	61 (20.1)
Underlying disease			
Yes	156 (58.2)	29 (82.9)	185 (61.1)
No	112 (41.8)	6 (17.1)	118 (38.9)

## Data Availability

The data used to support the findings of this study are available from the corresponding author upon request.
